# Diagnosis of Varroosis Based on Bee Brood Samples Testing with Use of Semiconductor Gas Sensors

**DOI:** 10.3390/s20144014

**Published:** 2020-07-19

**Authors:** Beata Bąk, Jakub Wilk, Piotr Artiemjew, Jerzy Wilde, Maciej Siuda

**Affiliations:** 1 Apiculture Division, Faculty of Animal Bioengineering, University of Warmia and Mazury in Olsztyn, Sloneczna 48, 10-957 Olsztyn, Poland; teofil.wilk@uwm.edu.pl (J.W.); jerzy.wilde@uwm.edu.pl (J.W.); maciej.siuda@uwm.edu.pl (M.S.); 2 Faculty of Mathematics and Computer Science, University of Warmia and Mazury in Olsztyn, Sloneczna 48, 10-957 Olsztyn, Poland; piotr.artiemjew@uwm.edu.pl

**Keywords:** bee brood, *Varroa destructor*, gas sensor, electronic nose, k nearest neighbor algorithm

## Abstract

Varroosis is a dangerous and difficult to diagnose disease decimating bee colonies. The studies conducted sought answers on whether the electronic nose could become an effective tool for the efficient detection of this disease by examining sealed brood samples. The prototype of a multi-sensor recorder of gaseous sensor signals with a matrix of six semiconductor gas sensors TGS 823, TGS 826, TGS 832, TGS 2600, TGS 2602, and TGS 2603 from FIGARO was tested in this area. There were 42 objects belonging to 3 classes tested: 1st class—empty chamber (13 objects), 2nd class—fragments of combs containing brood sick with varroosis (19 objects), and 3rd class—fragments of combs containing healthy sealed brood (10 objects). The examination of a single object lasted 20 min, consisting of the exposure phase (10 min) and the sensor regeneration phase (10 min). The k-th nearest neighbors algorithm (kNN)—with default settings in RSES tool—was successfully used as the basic classifier. The basis of the analysis was the sensor reading value in 270 s with baseline correction. The multi-sensor MCA-8 gas sensor signal recorder has proved to be an effective tool in distinguishing between brood suffering from varroosis and healthy brood. The five-time cross-validation 2 test (5 × CV2 test) showed a global accuracy of 0.832 and a balanced accuracy of 0.834. Positive rate of the sick brood class was 0.92. In order to check the overall effectiveness of baseline correction in the examined context, we have carried out additional series of experiments—in multiple Monte Carlo Cross Validation model—using a set of classifiers with different metrics. We have tested a few variants of the kNN method, the Naïve Bayes classifier, and the weighted voting classifier. We have verified with statistical tests the thesis that the baseline correction significantly improves the level of classification. We also confirmed that it is enough to use the TGS2603 sensor in the examined context.

## 1. Introduction

The hive air is a complex mixture of volatile substances, which can be identified by various methods [1]. So far, infrared analysers have been used, among others; Fourier transform infrared spectrometers (FT-IR spectrometers); or gas chromatographs with a flame ionization detector (FID), tuned mass spectrometer (MS) or other mass-selective detectors [2]. However, these methods are not practical for the average beekeeper and are not cost-effective.

A good solution in this case seems to be the use of e-noses, which are measurement systems based on a sensor matrix capable of identifying complex odours [3]. The sensors of volatile substances are classified in five categories. They differ in the operation mechanism, which is based on conductivity, electrochemical, gravimetric, optical, and thermal properties of the test sample. Semiconductor metal oxide sensors (MOS—metal-oxide-semiconductor) are most commonly used in the electronic nose, which work through the reduction oxidation reactions of adsorbed gases on the sensory layer [4]. The odour classification is then based on the values of the sensor readings expressing the sensor resistance [5,6]. On the other hand, by creating three parametric sensorgrams from measurements containing data about: maximum standardized sensor response, reaction time, and cleaning time, it is also possible to accurately determine the substances included in the tested gas mixture, as well as to give their concentration [7,8].

The most popular on the market at the moment, cheap and widely available are thick layer SnO_2_-coated sensors from Figaro (Japan) [9]. They are designed for continuous operation and are able to take measurements in dynamic mode. However, the most recent achievement is sensors based on a single SnO_2_ nanotube (SnO_2_ NWs sensors). They easily detect the presence of various gases and are able to estimate their concentration in the range 1–50 ppm. They are also successfully used to detect X-ray radiation [10,11,12].

The other modern solution in gas testing is to use sensors with layers applied to an array of capacitive interdigitated electrodes (chips). Such a solution allows to distinguish individual gas components, and in the case of Liquid Natural Gas (LNG) it can be used to calculate energy parameters. The sensitivity and selectivity of such a sensor is compared with a compact analyser using tunable infrared spectrometry with an infrared filter [13].

Electronic noses (e-noses), formed by a multi-sensor matrix of semiconductor gas sensors, have been used previously to recognize diseases in humans [14], animals [15], and plants [16]. They are so sensitive that they can be used to recognize the biotic causes of various diseases [17].

Varroosis is a dangerous bee disease. It is considered the main cause of bee colony collapse disorder (CCD) worldwide. This disease is caused by the parasitic *Varroa destructor* mite. His reproductive cycle occurs in cells with sealed brood, where it is invisible under the sealing. However, during the phoretic phase, the parasite hides under the abdomen of bees, which also hinders its detection [18]. Therefore, it is difficult to diagnose the invasion of *V. destructor* in bee colonies without complicated and detailed laboratory tests [19]. Therefore, veterinarians and beekeepers need tools to quickly detect varroosis.

In the case of bee diseases, electronic nose technology has never been applied to detect the pathogens themselves that threaten the bee colony. For varroosis, it was proposed to use a gas sensor system to detect residual formic acid used to combat *V. destructor*. This was to successfully monitor the effectiveness of this antivarroosis treatment [20]. Semiconductor sensors have also proved useful in diagnosing varroosis by direct examination of beehive air [6,21].

The second most dangerous bee disease after varroosis is American foulbrood. This disease also causes the death of bee brood. The American foulbrood is caused by the spore-bearing *Peanibacillus larvae larvae*. It seems that this bee disease can be successfully diagnosed with semiconductor gas sensors [22]. This is because the dying bee brood emits a specific smell resulting from the presence of caproic, valeric, and isovaleric acids [23]. Varroosis turns out to be a challenge for scientists. *V. destructor* mites that cause this disease entity are characterized by an odor mimicry, which consists in adapting the parasite odour to the host it is on [24]. Another bee disease worth mentioning is the Deformed Wing Virus (DWV). This virus is transmitted by *V. destructor* mites; therefore, bees often encounter deformed wings at the varroosis. The DWV virus brings additional changes to the volatile chemical fraction; [25] have shown that brood infected with this virus has a higher expression of such compounds as: 2- an 3-butanediol and 2- and 3-methylbutanoic acid. Thanks to these olfactory indicators, hygienic bees remove diseased brood more intensively [26]. It is therefore unlikely to show the presence of the mites themselves in the bee colony utilizing semiconductor gas sensors. However, high hopes are placed in fragrances created in bee brood infected by *V. destructor* [27]. Nazzi et al. [28] showed that short-chain unsaturated hydrocarbons, including alkene Z-6-pentadecen, play an important role in this process.

E-nose devices can be successfully used to demonstrate the presence of microbial pathogens [29], parasites [30], as well as changes in the metabolism of parasitized organisms [31]. It seemed, therefore, that the multi-sensor system could also work in diagnosing varroosis. Therefore, a prototype of a multi-sensor air quality detector with a 6-sensor matrix was created. The selection of sensors was not accidental. It was decided to use semiconductor metal oxide (MOS) sensors. They are not only sensitive to one target gas but also react to the presence of volatile organic compounds. At the same time, they are extremely sensitive at the ppm-level. They react quickly and regenerate and acquire data in real-time. Besides, these sensors are cheap, readily available, and can be easily integrated into integrated circuits. Their disadvantage is that they react to water vapor.

The study aimed to check whether a specific semiconductor gas sensor system can diagnose varroosis based on bee brood testing. An attempt was also made to answer the question of which sensors are most effective in this respect. It was checked whether the system was able to distinguish the degree of severity of this dangerous bee disease.

## 2. Materials and Methods

### 2.1. Construction of the Measuring Device

The measurements were carried out at the Bee Products Quality Monitoring and Safety Laboratory of the Apiculture Division of the University of Warmia and Mazury in Olsztyn (Poland). The measuring device was a prototype of a multi-sensor recorder of sensor signal with a matrix of 6 semiconductor gas sensors TGS823, TGS826, TGS832, TGS2600, TGS2602, and TGS2603 from FIGARO 9.

The idea of this gas sensor device, as well as the instrument itself, was developed in the Laboratory of Sensor Technique and Indoor Air Quality Studies, Wroclaw University of Science and Technology, Poland. The device is intended for testing to detect various bee diseases. It has been tested with good results in outdoor studies for the diagnosis of varroa [5,6]. The device is built of several functional modules. The core of the whole device is the MCA-8 multi-channel gas signal recorder. There is a matrix of all 6 TGS sensors. For each individual sensor used, there is a selective sensitivity to specific groups of volatile organic compounds (VOCs). With TGS2xxx series sensors having smaller detection ranges (from 1 ppm to dozens of ppm), the TGS8xx series detects substances between 10 ppm and 1000 ppm. Therefore, the sensors were selected in such a way that their sensitivity range to different groups of substances complement each other, giving a full view of the gas sample being tested (Table 1).

Since each sensor needs its own operating temperature, the individual sensors are placed in their own aluminium flow chambers. The size of the chambers dedicated for TGS8xxx sensors was 12 cm^3^ and for TGS2xxx sensors was 4 cm^3^. Such construction protected the sensors against interaction. The final effect of the work of our selected semiconductor sensors is not an indication of the quality or quantity of the analysed gas but an indication of its class membership. This is possible because these sensors give nonspecific reactions to various gas mixtures. This is because the sensors we have chosen work through oxidation reactions of reducing gases on their surface. This results in a resistance disturbance, which is converted into an electrical signal. When testing a gas sample, subsequent electrical signals are accumulated over time. Then the appropriate feature vector is formed, which allows each set of signals to be classified in the appropriate class.

**Figure 1:**
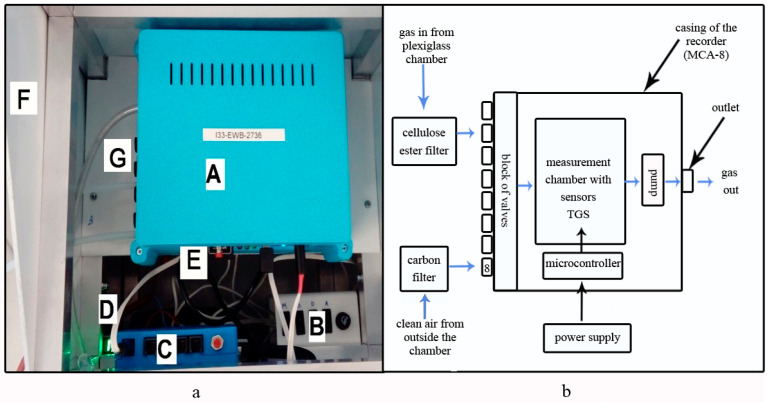
A prototype of the multi-sensor recorder of sensor signal based on semiconductor gas sensors: (**a**) General view: A - recorder (MCA-8), B - power supply control panel, C - control panel for data transfer to the server, D - panel power supply, E - card reader, F - housing, G - inlet channels; (**b**) block diagram with indicated directional gas flow.

The gas flow (sampling) is forced by a membrane pump with a maximum airflow of 5 L/min. The device has eight inlet channels and one outlet channel. The multi-sensor recorder is equipped with software from which you can control the pump power, heater power, sampling time, and change channels. The recorder has an SD card reader on which measurement data is saved every second. The next module is the Beecom communication controller. It allows the data from readings to be immediately sent to the server. All modules are in the housing (Figure 1).

### 2.2. Construction of the Measuring Stand

**Figure 2:**
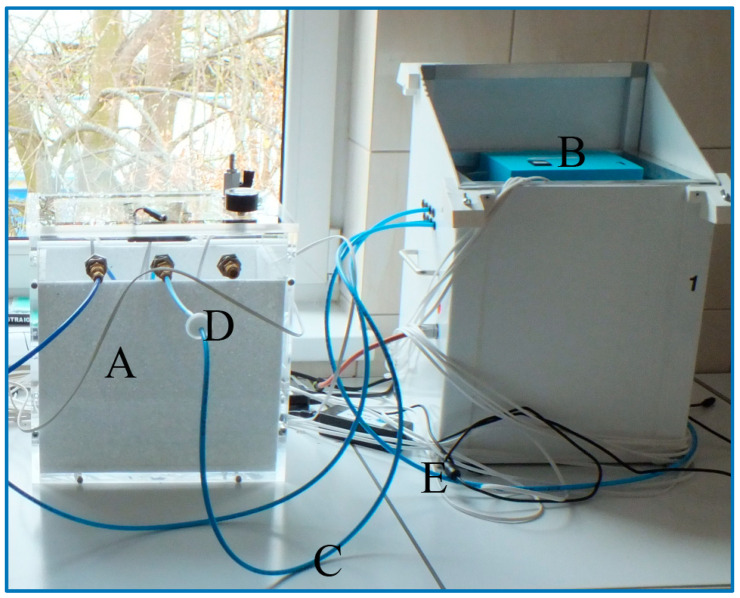
Measurement stand: A - chamber with polystyrene insert, B – a prototype of the multi-sensor recorder, C - pipe providing the gas from the chamber to the multi-sensor recorder, D - particulate and dust filter, E - pipe supplying clean air from outside the chamber for sensor regeneration.

The measuring stand was built from a prototype of a multi-sensor recorder of gas sensor sensing signals and a 32 cm × 22 cm × 32 cm plexiglass chamber. The chamber was lined with a polystyrene insert to imitate the conditions prevailing in the polystyrene hive. The chamber had sealed openings for the insertion of polyethylene (PE) tubes that performed a function of test probes. These tubes provided a gas sample taken from the chamber to the device through a specific channel, which was always, after two days of testing, changed from the position of the control software to the next one. There was a mixed cellulose ester filter (MCE 13 mm, 0.45 µm) in the conduit path that was to trap solid particles and dust. In the polystyrene chamber, the air was continuously replenished by the 0.5 cm diameter inlet in the upper part. This prevented the formation of negative pressure (Figure 2).

In the device, there was always a pipe supplying clean air from outside the chamber connected to the last channel 8. It was designed to regenerate the sensors. In this case, at the end of the test probe, there was a carbon filter that retained organic particles that could interfere with sensor readings.

### 2.3. Characteristics of Tested Brood Samples

Fragments of bee combs containing sealed brood, which consists of various stages of development of the bee: erect larvae, pre-pupae, and pupae constituted the samples of the brood. Bee brood samples were obtained from 19 bee colonies of the *Apis mellifera carnica* subspecies. Five bee colonies were healthy (on average 0% infected brood cells). Ten healthy brood samples were obtained from these colonies for testing (2 samples from each colony). Fourteen bee colonies were infected with the *V. destructor* parasite. The brood in these colonies was characterized by different levels of mite infestation (from 8.8% to 61.7%, Table 2). A total of 19 brood samples were obtained from these colonies. The study of the level of parasite infestation of brood samples was carried out after their testing with their multi-sensor device and the flotation method was used here [2].

**Figure 3:**
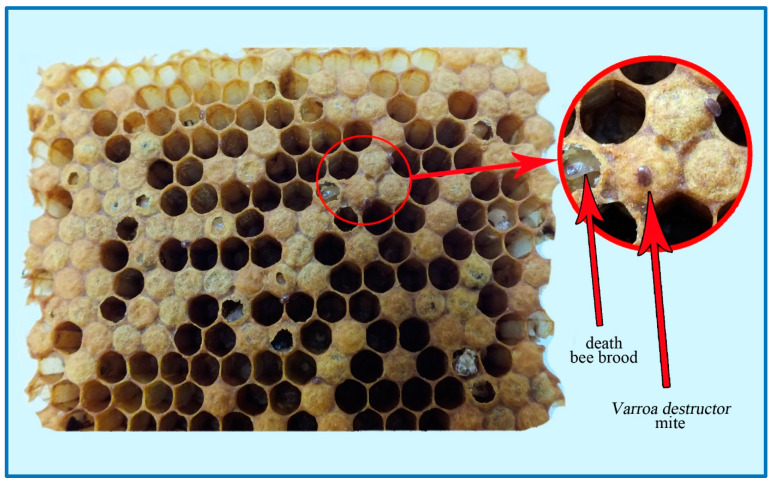
Sample of brood suffering from varroosis.

To obtain samples for research from the described colonies, whole combs with sealed brood were removed and brought to the laboratory. Fragments of sealed brood with an average surface area of 64 cm^2^ were cut out from the combs using a scalpel (Figure 3). The freshly cut fragment of a comb contained cut fragments of brood on the edges, which were removed with tweezers. Furthermore, the comb cells wet from leaking hemolymph were dried using an odourless paper towel. Then the samples prepared in this way were sent to the incubator (temperature: 35 °C) from where they were gradually removed for testing.

### 2.4. Class Characteristics

Three classes were created for comparison:

- 1st class—empty chamber (13 objects),
- 2nd class—fragments of combs containing brood sick with varroosis (on average 26.8% of infected brood cells) (19 objects),
- 3rd class—combs fragments containing healthy sealed (10 objects).

### 2.5. Characteristics of the Brood Subclass Depending on the Level of Infection by V. Destructor

To check how the readings of individual sensors were while the level of bee brood infestation with the *V. destructor* parasite increased in class 2, subclasses were distinguished:

- 2.1—highly infested brood (from 8% to 25%)
- 2.2—very highly infested brood (more than 25%) (Table 2).

### 2.6. Measurement Procedure

**Figure 4:**
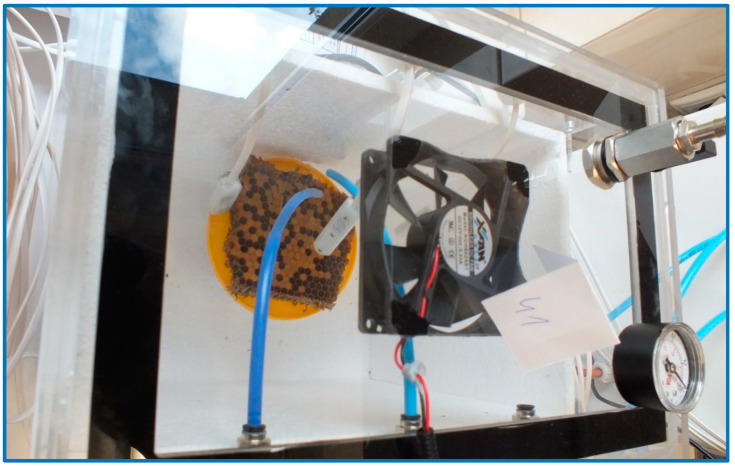
Healthy brood sample during the measurement.

The measurement procedure consisted of two phases: the sample exposure phase (10 min) and the sensor regeneration phase (10 min). During the examination of the gas sample from the empty chamber (exposure phase), the pump from the multi-sensor recorder sucked the air from the foamed polystyrene chamber (flow level at 0.5 L/min), which was supplied through a polyethylene tube (test probe) to the device channel and from there it went to the sensors. Tested gas sample was leaving the device through an outlet channel. Then clean air from outside the chamber was sucked in and transferred to the 8th channel of the recorder to regenerate the sensors (regeneration phase). The clean air was also leaving the device through the outlet. During the exposure phase of the sick and healthy brood samples, they were placed in the central part of the polystyrene chamber. Above them, at the distance of about 2–3 cm, the ends of the supply tubes (test probes) were located (Figure 4). The measurement took place. Then clean air outside the chamber was measured (regeneration phase).

### 2.7. Data Processing

**Figure 5:**
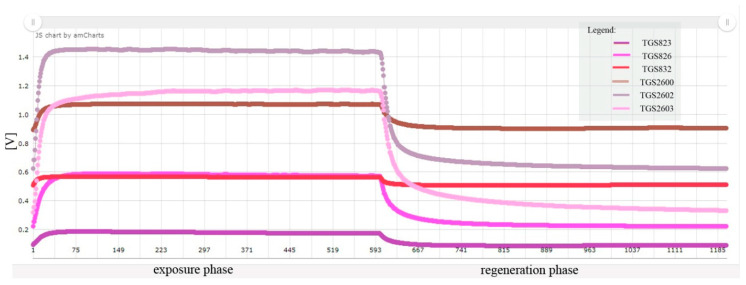
An example of a line graph for a measuring session of a single healthy brood sample.

Sensors, both in the exposure and regeneration phases, recorded measurement signals every second. A single measurement data corresponded to the average voltage recorded on the sensor in a period of 1 s before saving the measurement result. In total, 600 readings for the exposure phase and 600 readings for the regeneration phase were obtained during one measurement session. The measurement of the samples gave higher values of readings, which on the linear charts stabilized at a constant level at the latest after 250 s of measurement. After the sensors were exposed to the regeneration air, their reactions decreased; however, the reading value did not always return to the starting initial exposure, and the sensor readings stabilized at a constant level at the latest after 350 s of the regeneration phase (Figure 5).

To establish a common baseline for signals from gas sensors recorded during various measurements, a differential baseline correction was used.

The results were analysed in two variants:

- variant 1: 270 s sensor reading from sample measurement,
- option 2: value from 270 s of sensor reading from the sample measurement with baseline correction by subtracting the reading from the last 600 s of surrounding air measurement.

### 2.8. The Experimental Part Design

Due to the small number of samples available, a basic study of the class separation efficiency was carried out with results visualization. The efficiency of individual sensors, before and after the baseline correction, was compared.

Five times Cross Validation 2 (5xCV2) test was performed, which consists of dividing data in half and conducting two classifications, where each time different from two parts was treated as a training system. Cross Validation is used to assess how the results of statistical analysis generalize into independent datasets. Such a test allows us to calculate the average efficiency, the accuracy of which is shown by the variance of the results. The test allows us to determine how effective the model will be in practical application. The statistical features of this method can be seen in a detailed discussion [32] (page 57 and 58). The 5xCV2 allowed us to determine the average balanced accuracy of the classification. An important parameter is the true positive rate (TPR), which allows us to determine the precision of the classification (which is defined as the percentage of objects correctly classified in a class, of all objects classified in that class). As a reference classifier, the k nearest neighbor (k-NN) method was chosen with default settings (including automatically selected k) in the RSES tool (Rough set exploration system). The k-NN classifier is easy to implement and can be readily used by the measuring device data processing module. We have performed additional tests in the 5 times Monte Carlo Cross Validation 5 (5xMCCV5) model using set of additional, implemented by us, classifiers. In the experimental part we conducted a 1-tailed t-student test with the hypothesis that one series of results is better than another.

## 3. Results

### 3.1. Sensors Sensitivity to the Indicated Object Classes

**Figure 6:**
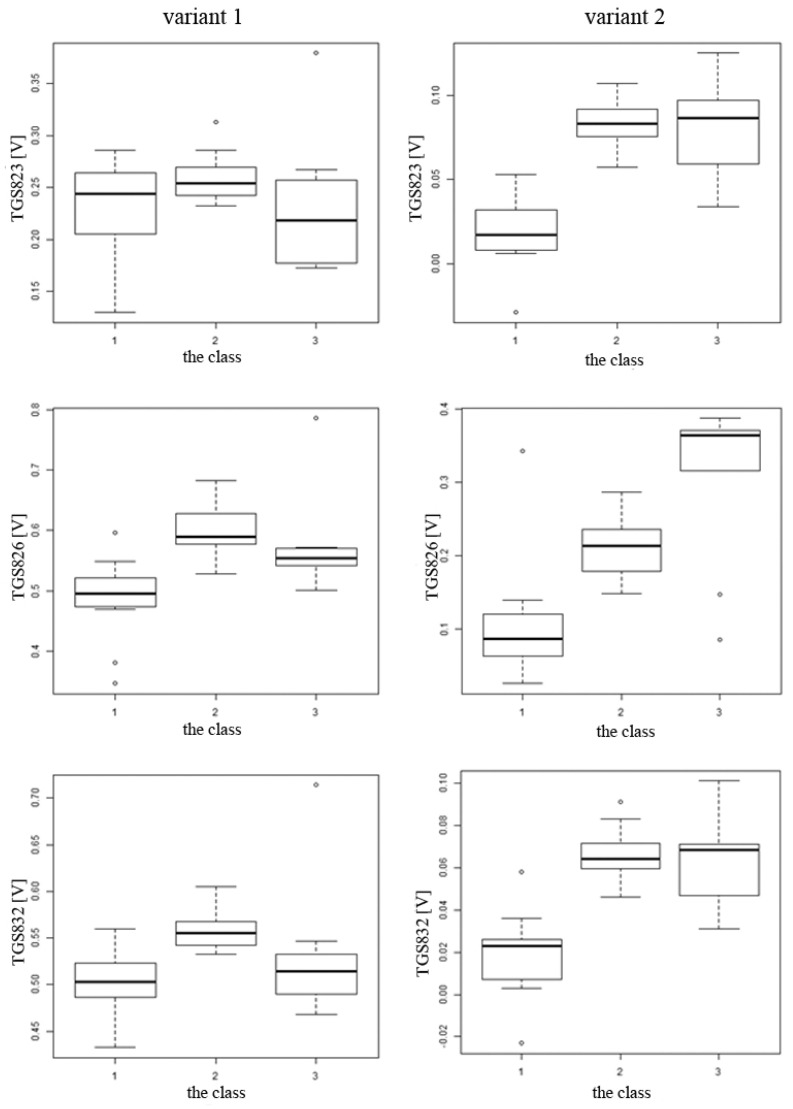

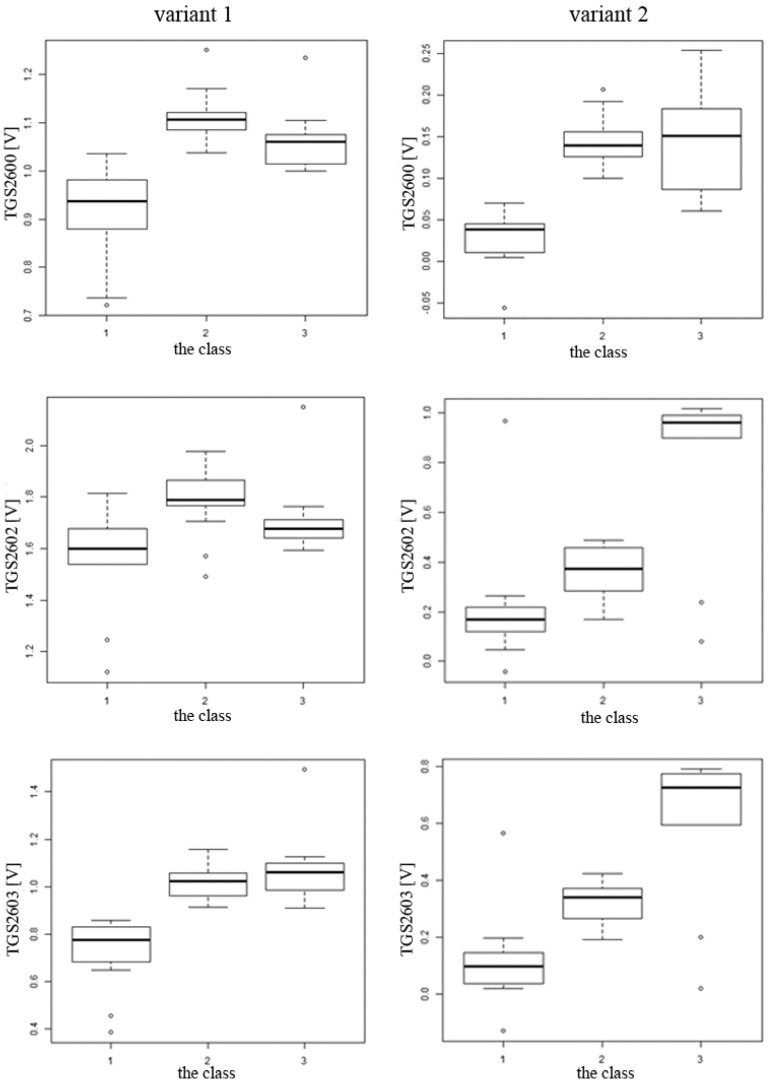
The average readings of individual sensors together with the value ranges for subsequent classes are presented comparatively for variant 1 (in 270 s of the sample exposure phase)—on the left and variant 2 (from 270 s of sensor reading from the sample measurement with baseline correction)—on the right. A clear improvement in the separability of individual classes after applying the baseline correction can be noticed.

The average readings for individual sensors along with the ranges of values for subsequent classes are presented comparatively for option 1 and option 2 in Figure 6. The initial visualization allowed us to notice that the analysis of the results according to option 1 does not allow us to identify which sensor is sensitive to the tested objects. It was found that the separability of individual classes improved after applying the baseline correction (option 2). This allowed the selection of the three most sensitive sensors—TGS 826, TGS 2602, TGS 2603—that effectively differentiate the classes of tested objects from each other.

**Figure 7:**
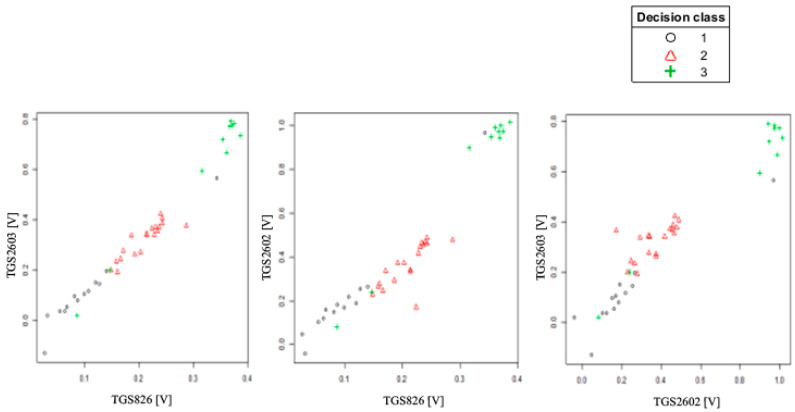
2D visualization of TGS826, TGS2602, TGS2603 sensor readings after baseline correction relative to each other.

**Figure 8:**
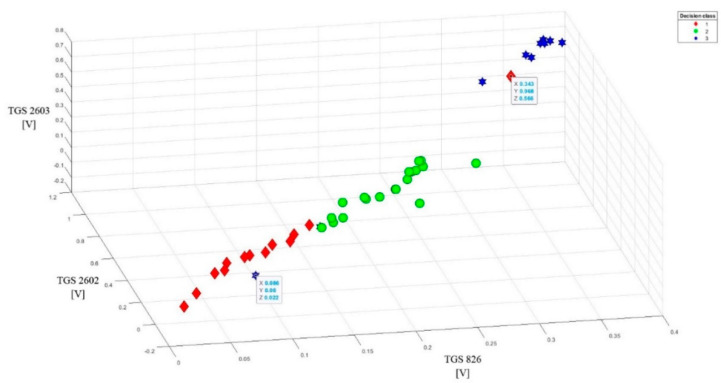
3D visualization of TGS826, TGS2602, TGS2603 sensor readings after baseline correction (3D visualization) relative to each other. Clear separation of individual classes.

To better present the effectiveness of these sensors in detecting brood sick with varroosis, visualization of the data after baseline correction (variant 2) was made in the form of points on the plane (2D visualization) (Figure 7). On three charts comparing readings from the most sensitive sensors in pairs, a clear separation of individual classes can be observed. 3D visualization (projection of points in three-dimensional space) of data after baseline correction enhances the effect of separating individual classes (Figure 8).

**Figure 9:**
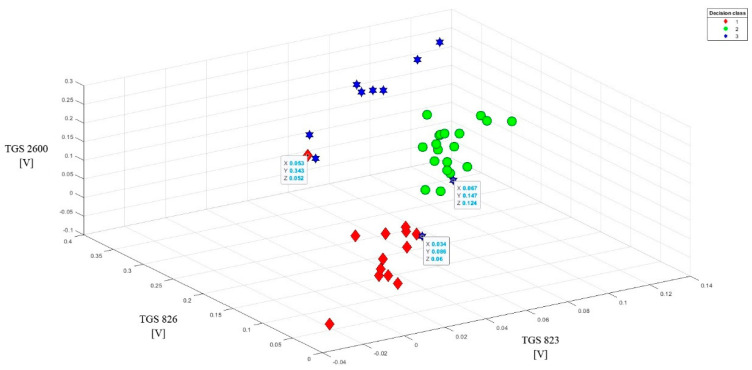
Sample data set after the baseline correction from the readings of two less sensitive sensors (TGS 2600 and TGS 823) with one more sensitive sensor (TGS 826) for the tested objects (3D visualization). High-class separability.

**Figure 10:**
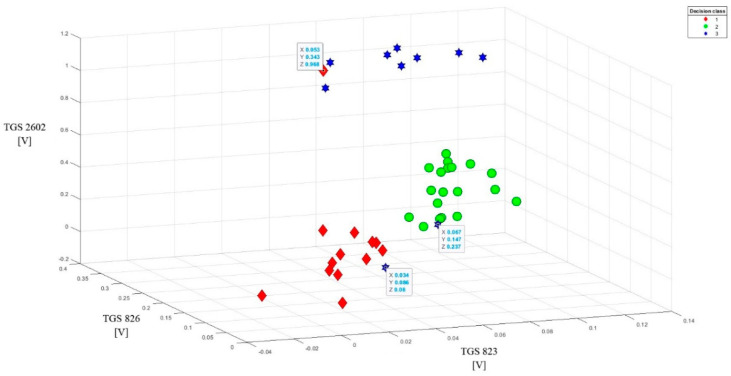
Sample data set after the baseline correction from the readings of one less sensitive sensor (TGS 823) with two more sensitive sensors (TGS 826 and TGS 2602) for the tested objects (3D visualization). High-class separability.

**Figure 11:**
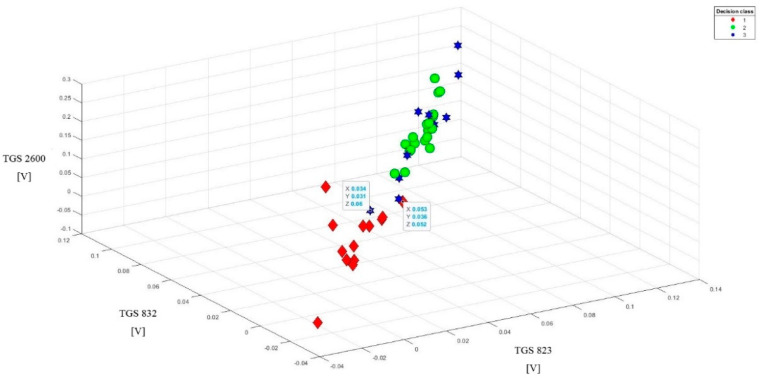
Data summary after baseline correction from three less sensitive sensor readings (TGS 823, TGS 832, TGS 2600). Poor class separability. Completely indistinguishable brood suffering from varroosis from the healthy brood.

This method of data presentation also allows showing sensitivity to the tested objects of other sensors, which in 1D and 2D visualization did not give such effects. We are talking about sensors: TGS623, TGS632, TGS2600. However, one condition must be met: readings from these sensors must be combined with one of the most sensitive sensors (Figure 9). Better effects in class separability are obtained when combining one less sensitive sensor with two more sensitive sensors (Figure 10). However, the data set from the sensors TGS623, TGS632, TGS2600 after correction on one chart does not give good separation of classes (Figure 11). This visualization showed that a matrix consisting only of these sensors would not be able to distinguish between brood suffering from varroosis and healthy brood.

The 3D visualization allowed us to detect three noisy data, obscuring the image. One object came from class 1 and two objects from class 3. They were listed on the charts and their exact parameters were in boxes. 

### 3.2. Analysis of the Results for the 5xCV2 Test

The mean accuracy of classification with usage of all sensors from the 5xCV2 model for 1st variant gives a global accuracy of 0.796 and a balanced accuracy (mean accuracy of all classes) of 0.757. The TPR for class 1 is 0.908, for class 2 is 0.772, and for class 3 is 0.768.

Using an analogous model for 2nd variant with baseline correction using readings from all six sensors, the average test results are as follows: global accuracy is 0.832, balanced accuracy is 0.834, class 1 accuracy is 0.836, class 2 is 0.92, and class 3 is 0.64. There is a clear gain in the average classification effectiveness for 2nd variant after baseline correction.

In addition, a similar experiment was carried out using only the readings from the TGS 2603 sensor in 2nd variant, which clearly best separates classes after baseline correction. It turned out that the results are comparable with those obtained for all sensors. Global accuracy is 0.841, balanced accuracy is 0.804, class 1 is 0.802, class 2 is 0.93, and class 3 is 0.77.

Without baseline correction, the classification with this attribute is significantly lower. Global accuracy is 0.741 and balanced accuracy is 0.717. The TPR for class 1 is 1, for class 2 is 0.7, and for class 3 is 0.48.

Thus, the adopted model of 5xCV2 indicates that the set of the semiconductor sensors used in the experiment allows detecting 92% of the diseased brood. Furthermore, the most sensitive TGS 2603 sensor itself shows similarly high accuracy in comparison with all six sensors (Table 3).

### 3.3. Analysis of the Results for the 5xMCCV Test

In the current test we check the efficiency of kNN classifiers (variants with the following metrics and parameters k: canberra.1nn, 2nn, 3nn, manhattan.1nn, 2nn, 3nn, euclidean.1nn, 2nn, 3nn) [32], Naïve Bayes Classifier (variant nb.num) [32], and 811 weighted voting classifier using selected metrics (variants canberra.811, manhattan.811 and euclidean.811) [32]. We present the results against the background of random classification (variant rand). The results can be seen in Table 4, Table 5, Table 6 and Table 7 respectively. To verify statistically, the thesis that baseline correction improves the level of classification, we have carried out a statistical test comparing a series of results from 25 subtests of the 5xMCCV5 method.

The results of the additional tests using a set of classifiers confirmed our earlier observation that the baseline correction improves the class separation results. Furthermore, the efficiency of classification can be maintained with even one sensor—TGS2603. To check if baseline correction gives statistically significant improvement in comparison with basic variant, we have used 1-tail t-student test. Let us move on to presenting this test on selected best results.

Please consider two series of balanced accuracy values, before and after baseline correction—a variant for TGS2603 sensor. A series of results were taken from the best method: Naive Bayes Classifier.

First series with baseline correction: {0.804233, 0.752137, 0.941176, 0.847222, 0.795238, 0.678571, 0.801587, 0.830688, 0.859259, 1, 0.944444, 0.865079, 0.477778, 0.924837, 0.894444, 0.944444, 0.663889, 0.790476, 0.829167, 0.790476, 0.833333, 0.844444, 0.793651, 0.726496, 0.916667}.

Second series without correction: {0.619048. 0.59127. 0.705556. 0.633333. 0.622222. 0.515152. 0.695437, 0.666667, 0.598214. 0.713889, 0.84127, 0.518519, 0.702614, 0.642857, 0.633333, 0.629167, 0.583333, 0.756944, 0.604762, 0.708333, 0.518519, 0.661905, 0.72619, 0.577778, 0.727778}.

There are 48 degrees of freedom, and critical t-value is 1.69 with significance level 0.05. Due to the fact that our calculated t-value (6.45) is greater than the critical value, we can say that the classification results are better significantly.

Let us run a similar test for the best result for all TGS sensors. We have used the kNN technique with Canberra metric, which turned out to be the best for examined data.

First series with baseline correction: {0.944444, 0.944444, 0.863095, 0.84127, 0.885714, 0.875, 0.805556, 0.896296, 0.833333, 0.766667, 0.814815, 0.861111, 0.792593, 0.822222, 0.888889, 0.847222, 0.958333, 0.847222, 0.833333, 0.952381, 0.896296, 0.866667, 0.916667, 0.792593, 0.888889}.

Second series without correction: {0.838095, 0.814815, 0.875, 0.688889, 0.547619, 0.768519, 0.891667, 0.60101, 0.763889, 0.833333, 0.811111, 0.842593, 0.761905, 0.625, 0.683333, 0.652381, 0.888889, 0.738095, 0.767677, 0.707071, 0.752137, 0.744048, 0.611111, 0.8, 0.888889}.

The calculated t-value 4.96 is greater than the critical value, hence the results are better after baseline correction to the degree of significance 0.05.

### 3.4. Distinguishing V. Destructor Invasion Levels in the Sick Brood

Since in the problem of classifying classes 2.1 and 2.2, we have a very small class 2.1 with seven objects only, we did not carry out the kNN classifications on these data. The analysis of sensor readings with baseline correction did not bring the expected results. The separability of the subclasses has deteriorated. Therefore, we have visualized the objects with their classes using selected combinations of three sensor readings without baseline correction. On such a small sample it is difficult to generalize, but selected 3D separation results have shown that most of the class 2.1 objects can be separated from class 2.2.

**Figure 12:**
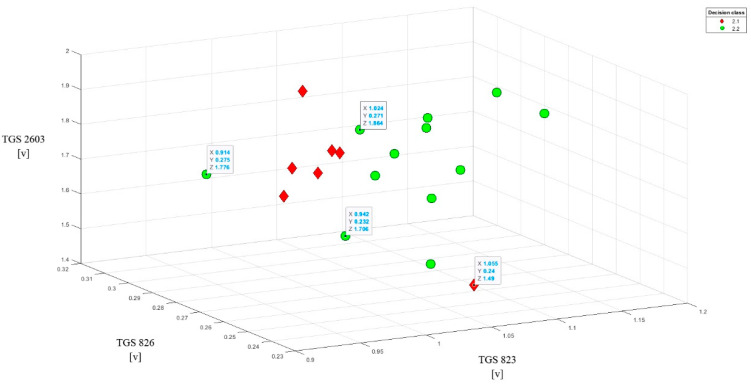
Data set from sensor readings (TGS 823, TGS 826, TGS 2603). Class 2.1 vs. 2.2. The graph shows the linear separability of most objects between classes, except for one object from each class.

An example of such separation is presented in Figure 12 showing the readings of the TGS 823, TGS 826, and TGS 2603 sensors relative to each other. It can be seen that in classes 2.1 and 2.2, two objects are outliers and enter the area outside of their classes. However, the remaining objects are grouped into linearly separable subgroups. Similar effects are obtained by combining the following sensor sets in 3D visualization: TGS 823 vs. TGS 2600 vs. TGS 2602; TGS 823 vs. TGS 826 vs. 2600, and TGS 823 vs. TGS 826 vs. TGS 832.

## 4. Discussion

The paper presents an innovative approach to the issue of varroosis diagnosing based on tests of sealed brood samples. Until now, the only recognized method for testing brood samples was the flotation method [19]. However, it requires the opening of brood cells, which is time- and labour-intensive and causes the death of developmental forms of bees. The use of semiconductor gas sensors can detect the presence of parasites in the brood under the sealing. However, after examining, the brood can be returned to the bee colony and will be successfully reared.

The results of the presented laboratory tests are preliminary. The device is to be used in the future to quickly diagnose varroosis even in field conditions. It is to be an indispensable tool for every veterinarian dealing with bee diseases. It will also serve the beekeeper, who cares for the health of his bee colonies.

The prototype effectiveness in diagnosing brood suffering from varroosis has been demonstrated after the baseline correction. This correction is recommended by many e-nose researchers [33,34,35]. This indicates the need to use this treatment in software algorithms that will support the final version of the device in the future. The present study used a different baseline correction based on the sensor cleaning (regeneration) process. However, other baseline correction techniques may not be excluded in the future.

The measurement procedure lasted 20 min: 10 min exposure phase and 10 min regeneration phase. The line charts obtained from the sensor readings based on visual analysis indicate that they stabilize at a certain constant level after 250 s (for the exposure phase) and after 350 s (for the cleaning phase) of the measurement, respectively. Thus, the duration of testing a single sample could be reduced to 10 min. This issue should be considered when implementing the software to the final version of the device, making calculations based on statistics seeking to determine the shortest possible measurement time guaranteeing satisfactory results.

The matrix of six sensors used in the prototype effectively recognizes bee brood suffering from varroosis. Relevance in this class was 0.92. The results show that if the prototype were to be used only to distinguish infected brood with V. destructor from a healthy one, then the matrix could consist of three sensors: TGS 826, TGS 2602, TGS 2603. However, if the detector were to be based only for economic reasons on one sensor, TGS 2603 would work great here, in which global accuracy, balanced accuracy, and accuracy in the class of sick brood and healthy brood coincided with the values for these parameters calculated for all sensors combined. By design, however, the prototype is intended to detect varroosis also by testing samples of bees or even entire bee colonies. In the latter case, the use of a single sensor in the diagnosis of V. destructor invasion proved to be insufficient and it is recommended to monitor the bee colony in this respect using the adopted matrix [5].

The classifier adopted in the tests turned out to be an effective tool for demonstrating the sensitivity of individual sensors to the tested objects. Class separability was obtained after baseline correction already at the 1D visualization level. Adding further dimensions of space deepened the effect of class separation. With 3D visualization, the juxtaposition of sensor readings previously insensitive to the tested objects with more sensitive sensors also allowed the separation of all attributes. The adopted classifier also proved effective in monitoring the invasion of V. destructor in bee colonies [5].

Three tested objects gave noisy data in the results. This issue was looked at more closely. It turned out that measurements for these objects were made on different days and on other objects than the presented classes. On those days, unusually fragrant objects were also measured outside the classes accepted in this work as part of other experiments. It seems, therefore, that this could be the reason for the darkening of the image. The right recommendation in such cases should be the extension of the sensor regeneration phase, as well as minimizing the diversity of external operating conditions of the instruments. The noise of some measurements indicates that the issue of the impact of environmental external conditions on the effectiveness of this type of measurement requires further targeted research.

Analysing the charts that illustrate the distance between class 2 subclasses, we concluded that a baseline correction does not give satisfactory separation of the subclasses, which may be because the correction in some cases brings objects of a given decision class closer together.

The monitored objects are research material of the same class and the correction applied is not very characteristic; it is not distinguishable for subclasses. The analysed data on levels of the parasite infestation of sick brood were characterized by small decision classes and the kNN classification was unreliable. In this case, 3D visualization of sensor readings without baseline correction in various combinations worked well. It allowed distinguishing different levels of parasite infestation of the sick brood. In this range, no sensor alone is effective. This, therefore, indicates the need for a sensor system. Therefore, studies have shown that the sensor matrix used in the MCA-8 device has great potential not only in recognizing the disease but also in determining its level of severity. This will allow in the future the end-user who will be a veterinarian or a beekeeper not only to react immediately to the presence of varroosis in bee colonies but also to choose appropriate methods of treatment for this dangerous bee disease. To confirm our basic results, we extended the experimental part with our own implementation of the kNN classifier with selected metrics, the Naïve Bayes classifier and variants of the weighted classifier. The best results, similar to the variant of the RSES system classifier, were obtained for the kNN method with the Canberra metric and for the Naïve Bayes classifier. The quality of the base line correction application was successfully verified with the 1-tailed t-student test.

## 5. Conclusions

1. To achieve a good separation of classes, it is necessary to perform a baseline correction. In this case, using a differential technique brings good effects. We verified the statistical validity of this hypothesis with a 1-tailed t-student test.
2. The k-NN, and in some cases Naïve Bayes algorithm, are excellent tools to demonstrate the sensitivity of the sensors used in the study to distinguish sick brood with varroosis from the healthy brood. If the number of objects was too small to perform a cross-validation test, 1D, 2D, and 3D visualizations were used.
3. The TGS 623, TGS 2602, and TGS 2603 sensors proved to be highly sensitive semiconductor sensors for the diagnosis of brood suffering from varroosis in the laboratory conditions.
4. The most effective sensor in distinguishing individual classes is the TGS 2603 sensor and it could be successfully used to diagnose varroosis in sealed brood samples.
5. The prototype of the MCA-8 multi-sensor gas sensor signal recorder makes it possible to distinguish perfectly from sick brood with varroosis from the healthy brood and it also distinguishes an empty chamber from brood samples.
6. The semiconductor sensor matrix used in the device makes it possible to differentiate *V. destructor* invasion levels in samples of the sick brood.

## Figures and Tables

**Table 1:** The characteristics of semiconductor gas sensors, which were used in the multi-sensor array [9].

Sensor	Substances Detected	Detection Range
TGS 823	Organic solvent vapours	50–5000 ppm Ethanol, n-Hexane, Benzene, Acetone
TGS 826	Ammonia	30–300 ppm Ethanol, Ammonia, Isobutane
TGS 832	Chlorofluorocarbons	100–3000 ppm R-407c, R-134a, R-410a, R-404a, R-22
TGS 2600	Gaseous air contaminants	1 ppm ~ 100 ppm
TGS 2602	VOCs and odorous gases	1–30 ppm Ethanol, Ammonia, Toluene
TGS 2603	Amine-series and sulfurous odour gases	1–30 ppm Ethanol 0.1–3 ppm Trimethylamine, 0.3–2 ppm Methyl mercaptan

**Table 2:** Level of brood infestation with the *V. destructor* for examined class 2 objects divided into subclasses.

Subclass of Infested Brood	Level of Brood Infestation with the *V. destructor* (min.–max.)	No. of Tests in a Class	The Average Level of Brood Infestation with the *V. destructor* in a Subclass
2.1	8.8–23	7	14.7
2.2	26.1–61.7	12	33.9

**Table 3:** Results of 5xCV2 experiment for 1st and 2nd variant—the reading from all sensors vs. TGS 2603.

	All Sensors—Readings from a 270-s of Sample (1st Variant)	All Sensors—Readings from a 270-s of Sample with Baseline Correction (2nd Variant)	TGS 2603—Readings from a 270-s of Sample (1st Variant)	TGS 2603—Readings from a 270-s of Sample with Baseline Correction (2nd Variant)
Global accuracy	0.796	**0.832**	0.741	**0.841**
Balanced accuracy	0.757	0.834	0.717	0.804
TPR for empty chamber	0.908	0.836	1	0.802
TPR for diseased brood	0.772	**0.920**	0.7	**0.93**
TPR for healthy sealed brood	0.768	0.640	0.48	0.77

**Table 4:** Average classification result for additional classifiers from 25 tests, 5xMCCV5 experiment, all sensors—readings from a 270-s of sample (1st variant).

classifier	acc_global_	coυ_globall_	acc_1_		acc_2_	acc_3_	acc_balanced_	tpr_1_	tpr_2_	tpr_3_
canberra.1nn	0.7824	1		0.711304	0.913432	0.609908	0.74488	0.956568	0.79886	0.614528
canberra.2nn	0.8176	1		0.712872	0.986208	0.56858	0.75588	0.974	0.790356	0.761332
canberra.3nn	0.7824	1		0.647612	0.994284	0.500572	0.714156	0.974	0.748012	0.786668
canberra.811	0.7216	1		0.509304	0.977144	0.375619	0.620692	0.947616	0.705676	0.621112
eps = 0.01.nb	0.6368	1		0.321304	0.906196	0.525808	058444	0.698668	0.73586	0.39269
euclidean.1nn	0.8096	1		0.774256	0.90938	0.631336	0.771656	0.90152	0.837768	0.668196
euclidean.2nn	0.8448	1		0.785164	0.954388	0.658004	0.799188	0.931236	0.837412	0.758
euclidean.3nn	0.8016	1		0.719504	0.94026	0.625244	0.761672	0.930284	0.803216	0.729576
euclidean.811	0.7152	1		0.604248	0.888168	0.435808	0.642748	0.987616	0.74638	0.392164
manhattan.1nn	0.8016	1		0.743364	0.908716	0.638004	0.76336	0.90852	0.833928	0.654956
manhattan.2nn	0.848	1		0.758748	0.97878	0.650004	0.79584	0.929568	0.825692	0.846668
manhattan.3nn	0.8128	1		0.710864	0.981856	0.581908	0.758212	0.929568	0.797872	0.839272
manhattan.811	0.7184	1		0.581612	0.913288	0.412096	0.635672	0.970616	0.755184	0.371046
nb.num	0.6224	1		0.329988	0.991788	0.156762	0.492844	0.716668	0.636364	0.402668
rand	0.3184	1		0.281342	0.345918	0.29019	0.305817	0.261002	0.511516	0.171499

**Table 5:** Average classification result for additional classifiers from 25 tests, 5xMCCV5 experiment, all sensors—readings from a 270-s sample with baseline correction (2nd variant).

Classifier	acc_global_	coυ_globall_	acc_1_	acc_2_	acc_3_	acc_balanced_	tpr_1_	tpr_2_	tpr_3_
canberra.1nn	0.8528	1	0.80596	0.945716	0.664336	0.7833	0.864648	0.929936	0.673572
canberra.2nn	0.8864	1	0.814692	1	0.707	0.815376	0.89498	0.907884	0.794188
canberra.3nn	0.8672	1	0.783388	1	0.676192	0.794668	0.90344	0.869868	0.731904
canberra.811	0.8256	1	0.619796	1	0.679288	0.74266	0.900808	0.804208	0.823616
eps = 0.01.nb	0.6864	1	0.498604	0.942092	0.379094	0.59002	0.751052	0.76514	0.431428
euclidean.1nn	0.8256	1	0.7232	0.934164	0.683384	0.757568	0.858104	0.892904	0.667568
euclidean.2nn	0.8688	1	0.7455	0.994668	0.734756	0.801268	0.89522	0.880092	0.767904
euclidean.3nn	0.848	1	0.663796	0.992	0.764048	0.782908	0.901028	0.838332	0.823616
euclidean.811	0.8448	1	0.65046	0.982416	0.76976	0.78014	0.84214	0.8386	0.8179
manhattan.1nn	0.8384	1	0.758692	0.943732	0.670048	0.76814	0.865092	0.90228	0.68076
manhattan.2nn	0.8848	1	0.768216	1	0.749756	0.81414	0.912312	0.885328	0.8179
manhattan.3nn	0.8656	1	0.714976	0.994668	0.758332	0.798956	0.901996	0.856032	0.816948
manhattan.811	0.8752	1	0.7293	0.991208	0.76976	0.806384	0.90676	0.87358	0.8179
nb.num	0.7872	1	0.869284	0.996924	0.140524	0.643728	0.873172	0.752716	0.54
rand	0.3264	1	0.276385	0.344204	0.316046	0.305917	0.282144	0.488192	0.205373

**Table 6:** Average classification result for additional classifiers from 25 tests, 5xMCCV5 experiment, TGS 2603—readings from a 270-s sample (1st variant).

Classifier	acc_global_	coυ_globall_	acc_1_	acc_2_	acc_3_	acc_balanced_	tpr_1_	tpr_2_	tpr_3_
canberra.1nn	0.726152	1	0.915344	0.76624	0.345143	0.675584	0.952228	0.749216	0.308132
canberra.2nn	0.75384	1	0.903008	0.862684	0.258667	0.674788	1	0.732404	0.265
canberra.3nn	0.747696	1	0.863632	0.86914	0.279667	0.670808	1	0.736044	0.304096
canberra.811	0.647688	1	0.62268	0.784052	0.308619	0.57178	1	0.651632	0.23719
eps = 0.01.nb	0.546152	1	0.347176	0.669696	0.615952	0.544276	0.86956	0.792708	0.238138
euclidean.1nn	0.727692	1	0.920344	0.76624	0.345143	0.677248	0.952228	0.749216	0.308132
euclidean.2nn	0.75538	1	0.906344	0.862684	0.258667	0.6759	1	0.733736	0.265
euclidean.3nn	0.750776	1	0.870964	0.86914	0.279667	0.673256	1	0.73876	0.304096
euclidean.811	0.698464	1	0.7363	0.81532	0.308238	0.619952	1	0.691932	0.225978
manhattan.1nn	0.727692	1	0.920344	0.76624	0.345143	0.677248	0.952228	0.749216	0.308132
manhattan.2nn	0.755384	1	0.910344	0.857728	0.258667	0.67558	1	0.738016	0.248413
manhattan.3nn	0.756924	1	0.878964	0.877744	0.263667	0.673456	1	0.742204	0.298868
manhattan.811	0.747696	1	0.863632	0.86914	0.279667	0.670808	1	0.691932	0.225978
nb.num	0.746156	1	0.875676	0.884952	0.182666	0.64776	0.9439	0.73978	0.242222
rand	0.34	1	0.34553	0.316154	0.358714	0.340133	0.328887	0.501964	0.18367

**Table 7:** Average classification result for additional classifiers from 25 tests, 5xMCCV5 experiment, TGS 2603—readings from a 270-s sample with baseline correction (2nd variant).

Classifier	acc_global_	coυ_globall_	acc_1_	acc_2_	acc_3_	acc_balanced_	tpr_1_		tpr_2_	tpr_3_
canberra.1nn	0.7408	1	0.515272	0.8687	0.720004	0.70132		0.602328	0.866416	0.622672
canberra.2nn	0.8016	1	0.548208	0.978312	0.696096	0.740876		0.74722	0.862592	0.658144
canberra.3nn	0.8064	1	0.570096	0.99498	0.665144	0.743408		0.84676	0.818036	0.658856
canberra.811	0.7936	1	0.475428	1	0.683432	0.71962		0.840288	0.790696	0.707716
eps = 0.01.nb	0.528	1	0.17146	0.588388	0.872188	0.544016		0.576668	0.892868	0.29373
euclidean.1nn	0.744	1	0.559208	0.863172	0.700576	0.707656		0.605824	0.871332	0.661072
euclidean.2nn	0.8512	1	0.712128	0.975644	0.723716	0.803828		0.79926	0.866788	0.88362
euclidean.3nn	0.848	1	0.720064	0.98208	0.699432	0.80052		0.856332	0.838548	0.809336
euclidean.811	0.8512	1	0.708004	0.97272	0.723428	0.80138		0.816	0.852464	0.876952
manhattan.1nn	0.744	1	0.559208	0.863172	0.700576	0.707656		0.605824	0.871332	0.661072
manhattan.2nn	0.8464	1	0.707128	0.967604	0.721912	0.79888		0. 779404	0.866348	0.87362
manhattan.3nn	0.856	1	0.717844	0.979224	0.733716	0.81026		0.82246	0.859196	0.889336
manhattan.811	0.8512	1	0.708004	0.97272	0.723428	0.80138		0.816	0.852464	0.876952
nb.num	0.8592	1	0.747684	0.947908	0.77038	0.821988		0.824248	0.884232	0.838288
rand	0.3536	1	0.369	0.345975	0.338476	0.351151		0.284929	0.571436	0.232509
